# Retraction: Amelioration of Aspirin Induced Oxidative Impairment and Apoptotic Cell Death by a Novel Antioxidant Protein Molecule Isolated from the Herb *Phyllanthus niruri*

**DOI:** 10.1371/journal.pone.0362436

**Published:** 2026-11-10

**Authors:** The PLOS One Editors

Following the publication of this article [1], concerns were raised with results presented in Figs 5-7. Specifically,

- The Fig 5 Phospho p38 and Phospho JNK panels appear more similar than would be expected from independent results, when one of these panels is rotated.
- The first lane and the last lane of the Fig 6A Bax panel appear more similar than would be expected from independent results, when one of these lanes is flipped.
- The Fig 7A ASA and Fig 7B ASA results appear more similar than would be expected from independent results.

The corresponding author stated they disagreed with the journal’s observations involving Figs 5 and 6. They stated they agreed the two ASA lanes in Fig 7 look similar and suggested that this may have been due to a data sorting error. The corresponding author stated that they are unable to retrieve the original raw data files. In the absence of the underlying data, these concerns cannot be resolved.

In light of the above concerns, which call into question the reliability and integrity of the published results, the *PLOS One* Editors retract this article.

PCS did not agree with the retraction. SB and SG either did not respond directly or could not be reached.

In addition to the above concerns, PLOS is aware of additional panel overlap concerns raised on PubPeer, involving panels presented in this article and panels published in other articles:

- Fig 4 Phospho NF-kB of [1] and Fig 5 NF-κB of [2]
- Fig 6B Phospho Akt of [1] and Fig 6 Bcl-2 of [3]
- Fig 6B PI3K of [1] and Fig 4A Bcl-2 of [4]
- Fig 7C Caspase-3 of [1] and Fig 7D Caspase-3 of [5]
- Fig 7C PARP of [1] and Fig 7D PARP of [5]

PLOS did not validate these concerns further as PLOS is unable to access [2–5] and the retraction of [1] is already warranted in light of the unresolved within-article concerns.

Furthermore, the Fig 5 Phospho p38 and Phospho JNK panels appear to partially overlap with the Fig 11A active Caspase-3 panel of [6, retracted in 7], which was published later by a different author group.
